# Developing recommendations for neonatal inpatient care service categories: reflections from the research, policy and practice interface in Kenya

**DOI:** 10.1136/bmjgh-2018-001195

**Published:** 2019-03-07

**Authors:** Claire Marriott Keene, Jalemba Aluvaala, Georgina A V Murphy, Nancy Abuya, David Gathara, Mike English

**Affiliations:** 1 Nuffield Department of Medicine, Centre for Tropical Medicine and Global Health, University of Oxford, Oxford, UK; 2 Kenya Medical Research Institute-Wellcome Trust Research Programme, Nairobi, Kenya; 3 Department of Paediatrics and Child Health, College of Health Sciences, University of Nairobi, Nairobi, Kenya; 4 Nairobi City County Government, Nairobi, Kenya; 5 School of Nursing and Midwifery, Aga Khan University, Nairobi, Kenya

**Keywords:** child health, health policy, health systems, paediatrics, public health

## Abstract

Neonatal deaths contribute a growing proportion to childhood mortality, and increasing access to inpatient newborn care has been identified as a potential driver of improvements in child health. However, previous work by this research team identified substantial gaps in the coverage and standardisation of inpatient newborn care in Nairobi City County, Kenya. To address the issue in this particular setting, we sought to draft recommendations on the categorisation of neonatal inpatient services through a process of policy review, evidence collation and examination of guidance in other countries. This work supported discussions by a panel of local experts representing a diverse set of stakeholders, who focused on formulating pragmatic, context-relevant guidance. Experts in the discussions rapidly agreed on overarching priorities guiding their decision-making, and that three categories of inpatient neonatal care (standard, intermediate and intensive care) were appropriate. Through a modified nominal group technique, they achieved consensus on allocating 36 of the 38 proposed services to these categories and made linked recommendations on minimum healthcare worker requirements (skill mix and staff numbers). This process was embedded in the local context where the need had been identified, and required only modest resources to produce recommendations on the categorisation of newborn inpatient care that the experts agreed could be relevant in other Kenyan settings. Recommendations prioritised the strengthening of existing facilities linked to a need to develop effective referral systems. In particular, expansion of access to the standard category of inpatient neonatal care was recommended. The process and the agreed categorisations could inform discussion in other low-resource settings seeking to address unmet needs for inpatient neonatal care.

**Summary box**

- There has been an absence of strategic thinking on the development of inpatient neonatal services in Kenya. This is likely to limit provision of essential interventions at scale as part of efforts to improve newborn survival.
- Presentation of collated evidence to stakeholders, with whom the research group had a long-standing relationship, and the use of a modified nominal group technique to facilitate consensus, was a productive and acceptable approach to drafting health service recommendations in the Kenyan context and might usefully be replicated in other settings.
- Consideration of the tensions surrounding choices, clarifying the perspective to be adopted and a focus on pragmatism helped achieve most decision-making goals within achievable time constraints. However, there was some tension between pragmatic and aspirational recommendations that led to a lack of consensus on certain services, and efforts to promote consensus-based decision-making did not entirely overcome the potential influence of high-status experts.
- Embedding research groups within health policy and delivery systems helps develop understanding of the context and the ability to facilitate evidence informed decision-making discussions. However, researchers can also introduce their own biases, making transparent reporting of processes important.

## Introduction

As targeted interventions reduce the prevalence of common causes of childhood death (eg, diarrhoea and pneumonia), newborn deaths contribute a growing proportion of under-five mortality.^1^ Estimations suggest that effective delivery of essential newborn interventions could decrease neonatal mortality by 71%, and that 82% of this reduction could be achieved by optimising delivery of inpatient care.^2^

Improving the organisation of inpatient newborn services is a major health system concern, particularly in low-income and middle-income countries (LMICs) such as Kenya, where neonatal mortality is high. Regionalisation of services, with concentration of resources at higher levels, has been suggested to improve service quality and efficiency and maintain staff skills.^3–5^ This centralisation of services relies on a well-functioning referral network; otherwise, it loses its benefits and its disadvantages are compounded.^6^ However, the Kenyan referral system currently faces many challenges, including a lack of written policy on transport logistics and financing, no coordination structure to oversee the implementation of the national referral strategy and a lack of quality standards and monitoring of referral service performance.^7^ Alternatively, decentralisation of services to lower-level facilities may reduce the burden on overcrowded higher-level facilities, increase access and improve responsiveness to local needs.^8–10^

Since the *Toward Improving the Outcome of Pregnancy* report in 1976, regionalised, hierarchical systems of newborn inpatient care have predominated.^3^ However, a 2014 systematic review found that evidence originated only from high-resource settings and was mostly of poor quality, making it insufficient to make causal claims on the relationship between regionalisation and the improvements seen in perinatal outcomes.^1^ Research into health systems is inherently complex and the implementation of regionalisation coincided with other improvements likely to influence neonatal outcomes in higher-income settings (generalised improvement in socioeconomic status, the introduction of new treatments and overall investments in health systems) and overlapped with a global decrease in neonatal mortality that transcended different system structures.^1 11^ This lack of quality evidence to guide structural organisation of neonatal inpatient systems is a barrier to service improvement, particularly in resource-constrained settings.

## Context

The Kenyan health system is hierarchical with community-based care at level 1 and facilities arranged from dispensary clinics and health centres (levels 2 and 3, respectively) to tertiary hospitals (level 6). County hospitals (formerly district hospitals), where much of the inpatient newborn care is provided, form levels 4 and 5.^12^ Emergency Obstetric and Neonatal Care (EmONC) categories have been integrated into the thinking on levels of care in Kenya: basic EmONC should be provided in level 2 and 3 facilities, and comprehensive EmONC from level 4 and above.^13^ However, there has been no prior attempt to categorise inpatient neonatal services for sick babies that might complement policy on levels of care in Kenya.

Our work focuses on Nairobi City County, where more than half the 4.26 million inhabitants live in low-income areas.^14^ Neonatal mortality is almost double the national average (39 compared with 22 deaths per 1000 live births), despite 89.4% of women delivering in a facility (compared with 42.6% nationally).^15^ In prior work, we have estimated that 44.5% of sick newborns likely to require admission do not reach one of the 31 facilities providing 24-hour inpatient neonatal services, and an additional 30.4% access inadequate inpatient services.^16 17^ Importantly, only 4 of these 31 facilities are in the public sector (three level 4/5 and one level 6 hospital), but they are responsible for 71% of the newborn admissions in the county, resulting in severe overcrowding and low nurse:patient ratios.^15 17^

Governance of health service delivery in Kenya was devolved to county level in 2010; however, work in the Nairobi context has implications for Kenya as a whole, with policy and capacity building remaining the responsibility of the national government.^18 19^ The national Ministry of Health has prioritised strategic development of public neonatal services to improve access and quality of care for newborns, particularly necessary for low-income groups that typically rely on the public sector.^20^ Previous work revealed a lack of standardisation of newborn inpatient services and the absence of formally agreed referral systems in Nairobi, thus technical guidance on the scope of newborn services that should be offered by facilities could help inform strategies for service expansion.^14^

The first step to address the need for expansion of services and improvement in quality was to develop recommendations defining categories of neonatal inpatient services applicable to the different facility levels in Nairobi City County. To achieve this, we set out to critically review existing Kenyan policy documents and normative guidelines to identify and incorporate existing recommendations relevant to newborn services, and identified examples of efforts to define categories of newborn inpatient care applicable to lower resource settings that could inform Kenyan discussions. We used this evidence to engage key stakeholders in order to develop draft recommendations defining categories of neonatal inpatient care, drawing on their experience and context knowledge.

## Approach

### Critical review of existing Kenyan policy and normative guidelines

We examined 12 documents spanning national strategic plans, clinical guidelines, human resource recommendations and quality of care policies (online supplementary appendix table A1). Policy documents support improved newborn care as a priority in the Kenyan health system strategy. They define general services to be provided at each level of care along with overall workforce profiles, but make specific reference only to basic immediate newborn care and care at delivery rather than inpatient care of small or sick newborns.

10.1136/bmjgh-2018-001195.supp1 Supplementary data

Although EmONC categories are well developed, of their nine signal functions only one is relevant to newborns: the ability to perform basic neonatal resuscitation.^21^ In the Kenyan public sector, basic EmONC should be provided from level 2 and comprehensive EmONC (including the capacity to provide caesarean operative deliveries) from level 4. Typically only level 4 to 6 facilities offer 24-hour neonatal inpatient care in the public sector, and there are only four of these facilities in Nairobi.^21 22^ There is no specification of what, if any, newborn inpatient care should be provided at levels 2 and 3 (where many deliveries are currently conducted) and no specific standardisation of neonatal service requirements at level 4, 5 or 6.

### Evidence-informed decision-making

Governments are encouraged to develop evidence-based policies with support from technical departments, advisers, academic and research groups.^23^ Many high-income countries have invested significant resources in this process, such as the National Institute for Health and Clinical Excellence in the UK.^24^ Such institutions do not yet exist in many LMICs, with guidance usually derived from recommendations from WHO or its partners such as the United Nations International Children’s Emergency Fund (Unicef). By their nature, these organisations focus on global rather than country-specific priorities and require local adaptation.^25^ This process can be directed by the accompanying WHO guidelines on adaptation and should consider contextual factors such as cost, local values, preferences and feasibility, which influence policy as much as evidence does.^24^

#### Examples of efforts to define newborn care services

High-income settings have extensive and granular guidelines on service provision by category of newborn care. Examples include the American Academy of Pediatrics’ guidelines that suggest four additive categories of neonatal care,^5 26^ or the National Health Services and British Association of Perinatal Medicine’s recommendation of the services that should be offered at three categories of newborn care in the UK.^27–32^ However, these are not easily translated to the Kenyan context. WHO and Unicef also do not currently have generic global guidance on how neonatal services should be structured and organised into categories.

Efforts have been made to categorise certain services into levels of care, such as the basic, special and intensive categories outlined by the BMC Pregnancy and Childbirth series.^33–35^ We also identified experience from LMIC settings through discussions on the Child Health Information for All forum and contacts in newborn health system development. Those felt to be most relevant to Nairobi City County were from India, on the development of Special Care Newborn Units (table 1),^36–40^ and South Africa, on the scale-up of care through the Limpopo Initiative for Newborn Care (LINC) (table 2).^41–43^ Both projects were supported by Unicef and produced toolkits categorising services and providing guidance on the staffing, infrastructure and unit size requirements (online supplementary appendix tables A2–A3).

**Table 1:** Division of neonatal care in India into three categories of inpatient care and one level of routine care associated with four facility levels^36–39^ (details in online supplementary appendix table A2)

Category of care	Facility level and package of care
Newborn care corner	**Primary health centres and hospitals:** All newborns Neonatal resuscitation and routine care at birth Identification and referral of at-risk or sick newborns Care provided by an auxiliary nurse midwife
Inpatient care	
Neonatal stabilisation unit	**Level 1 (community health centre):** Management of uncomplicated small neonates (>1800 g/>34 weeks’ gestation) Management of jaundice (phototherapy) and sepsis Care provided by a minimum of nurses (≥1) and medical officers (MOs) or paediatricians (≥1)
Special care newborn unit	**Level 2 (district hospital):** Management of small neonates (1200–1800 g/30–34 weeks’ gestation) Management of sick neonates ≥1800 g with birth asphyxia, meconium aspiration, jaundice (exchange transfusion), sepsis or requiring gavage feeding Care provided by a minimum of nurses (1 nurse:1.2 neonates), MOs (1 MO:4 neonates) and paediatricians (≥1)
Neonatal intensive care unit	**Level 3 (tertiary hospital):** Management of small neonates (<1200 g/<30 weeks’ gestation) Mechanical ventilation and surgery Care provided by a minimum of nurses (1 nurse:1 neonate) and neonatologists

**Table 2:** Division of newborn care by the LINC project into six categories of care combined into newborn units across the five South African facility levels^41–43^ (online supplementary appendix table A3)

Category of care	Facility level and package of care
Routine care	**Primary healthcare (clinic) and hospitals:** All newborns Neonatal resuscitation and routine care at birth Identification and referral of at-risk or sick newborns Care provided by enrolled and professional nurses
Inpatient care	
Kangaroo mother care	**Level 1 (district hospital), 2 (regional hospital) and 3 (tertiary hospital):** Management of uncomplicated neonates <2000 g Provision of warmth, nutrition and infection prevention
Standard inpatient care	**Level 1, 2 and 3 hospitals:** Management of small neonates (1500–2000 g/32–36 weeks’ gestation) Management of large neonates (>4000 g) Management of jaundice (phototherapy), possible infection, wasting, low Apgar scores, meconium staining, uncomplicated congenital abnormalities and neonates requiring oxygen or gavage feeding Care provided by a minimum of professional nurses (1 nurse:6 neonates), enrolled nurses and medical officers (MOs) (24-hour care if >18 beds)
High care	**Level 2 and 3 hospitals (may be provided at level 1 depending on available referral centres):** Management of small neonates (<1500 g/<32 weeks’ gestation) Management of jaundice (exchange transfusion), sepsis, convulsions, meconium aspiration, recurrent apnoea, moderate to severe respiratory distress requiring >40% oxygen with a head box, nasal prong CPAP or short-term intermittent positive pressure ventilation Management of chest drains and simple surgical conditions Care provided by a minimum of professional nurses (1 nurse:2 neonates), enrolled nurses and MOs (24-hour care if >18 beds)
Intensive care	**Level 2 and 3 hospitals** Management of complex or multisystem medical conditions, persistent hypoglycaemia, cardiovascular disease and neonates requiring invasive ventilation, total parenteral nutrition, therapeutic cooling or advanced neurological or cardiovascular monitoring Management of surgical conditions Care provided by a minimum of professional nurses (ideally 1 nurse:1 neonate; however, it is acceptable to have a ratio of 1:2 at level 3 and 1:3 at level 2), MOs, paediatricians and neonatologists (at level 3)
Specialised care and surgery	**Level 4 (quaternary hospital):** Specialised medical and surgical services spanning provincial boundaries Care provided by a minimum of professional nurses (ideally 1 nurse:1 neonate, but acceptable to have a ratio of 1:2), MOs, paediatricians (full time) and neonatologists

CPAP, continuous positive airway pressure; LINC, Limpopo Initiative for Newborn Care.

### Development of draft recommendations

The collation of evidence was completed in 5 months, producing a contextualised report of key findings (a format previously shown to be preferred by local decision-makers^44^). After examination of international recommendations, Kenyan guidance and the literature, key services and interventions were identified by the researchers as reasonable expectations of the public sector. Clear Kenyan guidelines exist for neonatal resuscitation and basic immediate newborn care (such as immediate thermal care, hygienic cord care and initiation of breast feeding),^20 45–47^ thus it was assumed these would be available in any facility where deliveries were performed (including those not providing inpatient neonatal care) and these services were not included in the panel discussions. These were further refined and omissions identified in collaboration with local paediatricians and nursing professionals, to produce a list of 38 services for categorisation by workshop participants (online supplementary appendix table A4).

We drew on the SUPPORT framework, within the limits of available resources, to present the evidence in this report. The framework is a structured approach designed to incorporate evidence into broader policy decisions and recommends a stepwise process of clarifying the problem, assessing options, choosing an intervention and planning for implementation.^48^ Ideally, framing options for presentation to decision-makers should cover the benefits, harms, costs and potential acceptability of each.^49^

A group of experts was identified for a meeting to draft initial recommendations on the categorisation of these newborn services. Stakeholder engagement built on the research team’s prior experience in creating national paediatric guidelines and a long-term process of research engagement to improve newborn care in Nairobi City County, which included formal stakeholder mapping,^50^ estimation of the burden of newborn care requirements^14 15^ and multiple advisory group meetings.^51^ Nine representatives from key stakeholder groups took part in the meeting (online supplementary appendix table A5). They were identified from the stakeholder analysis, including frontline clinicians, representatives of county and national government, professional associations and academic institutions.^50^ Many of the participants had contributed to previous expert group advisory meetings^15 51^ helping to build a culture of evidence-informed decision-making.^44^

The contextualised report was sent to the group in advance of the meeting, which was facilitated by a senior researcher and the main author of the report. Key findings were presented at the start of the meeting followed by opportunities for questions to create a common understanding of the evidence and its limitations. The approach to building consensus on the categorisation of these services, including the use of modified nominal group techniques, is described in box 1, which also describes the subsequent development of proposals on minimum staffing requirements.

Box 1 **Description of the workshop to categorise neonatal inpatient services in Nairobi City County**

**1** **-** **day workshop aiming to answer:**

- What services should each of the three categories of neonatal inpatient care provide?
- Which healthcare workers, and in what numbers, should be present to provide the services for each category?

Introduction of the panel to the task through a 45-minute structured PowerPoint presentation

- Clarification of the problem
- Presentation of collated evidence (complementing the written summary provided prior to the workshop)
- Presentation of tensions and considerations for the Nairobi City County context, including the research group’s estimates of the demand for services
- Facilitated discussion to ensure understanding of the background information and the group’s task, as well as an opportunity to highlight any obvious omissions in the list of services

**Figure 1:**
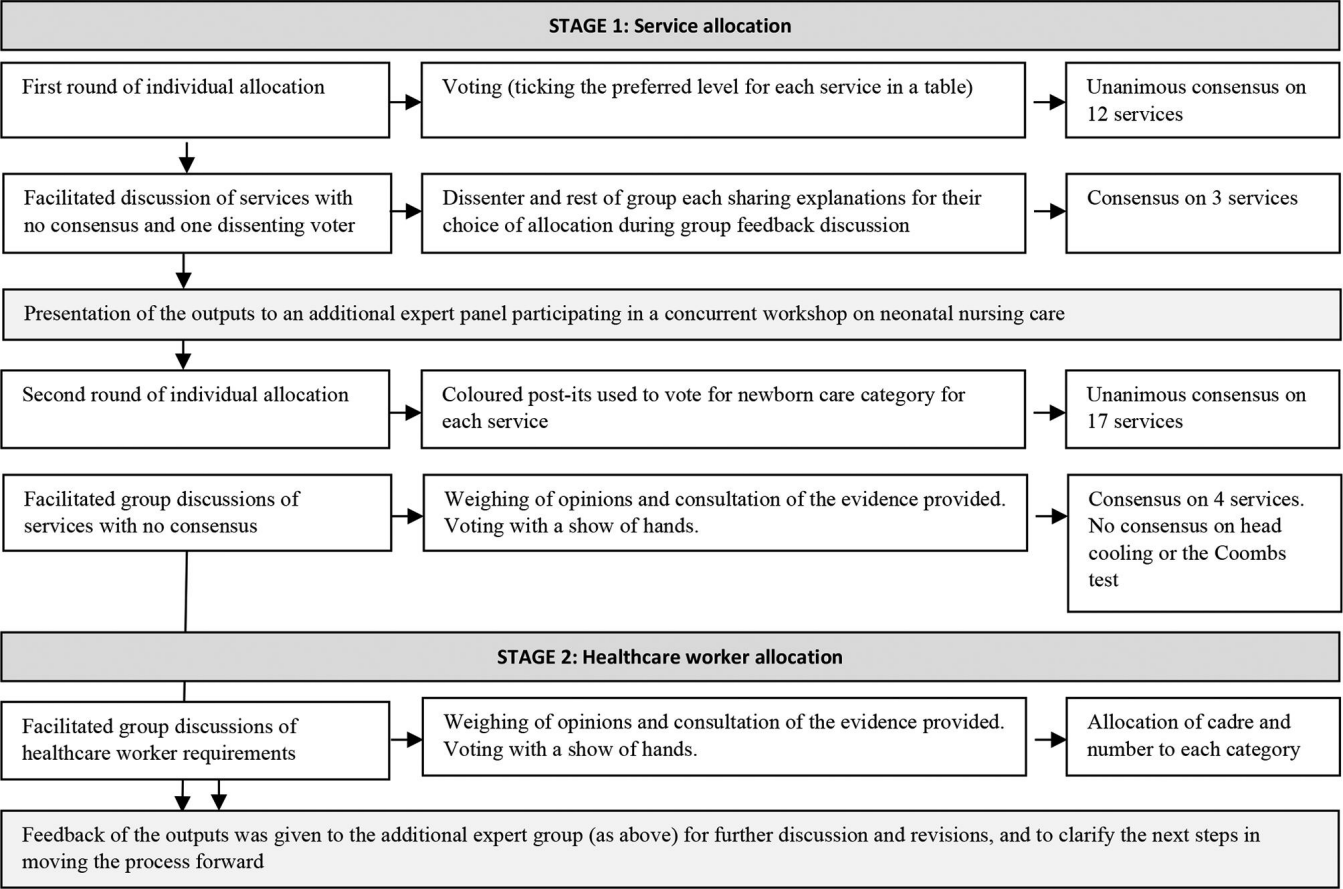
Workshop process, using a modified nominal group approach, to allocate proposed services and healthcare worker cadres to three categories of neonatal inpatient care.

Group process including modified nominal group approaches (see figure 1. For more details, see online supplementary appendix table A6):

- The panel agreed an overarching approach to developing guidance and assumed a pragmatic 5-year horizon
- For the allocation of services, we adapted the nominal group technique, which has been shown to be superior to individual problem-solving for questions that require judgement. Throughout the workshop process, participants were given space to think about the identified problem, individually make a decision and record this through various methods of voting. This ensured that each participant’s opinion was taken into account and prevented domination of one group member’s opinion in the results, as a greater number of diverse inputs improve decision quality. Multiple rounds of polling also encouraged more equal participation. Individuals then had the opportunity to provide the reasons for their choices during facilitated group discussions between rounds of voting^52^
- Unanimous allocation to a specific category of neonatal care was considered consensus and that service was not further discussed. Facilitated discussions on the remaining services were held, with the provision of further evidence on the services (benefits and impact, potential for adverse effects, cost, skill and technical requirements) and discussions of the case examples and WHO recommendations. If those dissenting to the majority allocation agreed that they understood the group decision and assented to having that service be allocated to the category the majority felt was appropriate, consensus was also considered achieved. The WHO recommendations were used as a pivotal point of information when services were unable to be categorised after considering the available evidence. If dissenting participants felt that the majority was wrong or that guidance was insufficient, no consensus was reached.

#### Framing of the discussion

Before addressing possible recommendations, the group discussed the tension between producing aspirational and pragmatic guidance, the constraints in existing infrastructure, workforce, transport networks and other resources, and the stage of development and availability of allied services (such as physiotherapy). The group’s previous research estimating the demand for services was also presented to contextualise the gaps in service coverage and highlight the need for expansion of services and improvement of quality. The group chose to focus on shorter-term goals that consider the current constraints faced by the health system rather than aspirational guidance.

The participants recognised that despite variation, global recommendations typically employ three major categories of neonatal services (low, intermediate and high) rather than finer gradations and focused on defining these three categories. Furthermore, a clear separation was made between the concept of ‘categories’ of neonatal services (provided by a specific unit within a facility) and the health system ‘levels’ that define these facilities in Kenya.

The group adopted the conventional approach of defining categories by specifying the package of services and interventions that each category should provide. These categories form an additive hierarchy so that a unit designated any specific category would typically also be expected to provide services allocated to lower categories. Individual units are defined by their highest category of care, which then dictates expected staff, equipment and infrastructure requirements. The participants were asked to individually allocate the 38 identified services and interventions to one of three service categories, named for the Kenyan context as standard, intermediate and intensive categories of neonatal inpatient care.

## Outcomes

The outcomes of the group’s deliberations are presented in tables 3 and 4.

**Table 3:** Services defining each category of neonatal inpatient care (it is assumed that facilities providing more advanced categories of care also provide all services of lower categories in an additive hierarchy)

Category of newborn inpatient care	Services and interventions allocated to each category by the expert group	
Standard care	Chest X-ray Thoracic transillumination Peripheral line Intravenous fluids Nasal prong oxygen Intravenous antibiotics Anticonvulsants (intramuscular phenobarbitone) Phototherapy	Kangaroo mother care Nasogastric tube Full haemogram Bedside glucose Laboratory glucose Total bilirubin Direct bilirubin Urea, electrolytes, creatinine
Intermediate care	Cranial ultrasound Upper/lower gastrointestinal barium X-ray CT scan/MRI scan Umbilical line Peripherally inserted long line Continuous positive airway pressure Surfactant Transfusion of blood (packed red cells)	Transfusion of blood products (fresh frozen plasma/platelets) Tube thoracostomy Double phototherapy Exchange transfusion Parenteral nutrition Retinopathy of prematurity screening (for referral to specialist care for management) Coombs test. The expert group was divided in allocating the Coombs test to intermediate or standard care, and no consensus was reached. At a minimum, we suggest that the Coombs test is provided in the intermediate category of care. Further information on the discussions on the Coombs test is provided in online supplementary appendix table A7
Intensive care	Arterial line Invasive ventilation Inotropes Peritoneal dialysis	Surgical management of gastroschisis, imperforate anus and necrotising enterocolitis Head cooling. The group was divided in allocating head cooling to intensive or intermediate care, and no consensus was reached. At a minimum, we suggest that head cooling is provided in the intensive category of care. Further information on the discussions on head cooling is provided in online supplementary appendix Table A7

**Table 4:** Minimum healthcare worker complement allocated to each category of newborn inpatient care and the numbers required to provide services (in brackets)

Standard care	Intermediate care	Intensive care
Dedicated to the newborn unit		
Medical officer OR Paediatric clinical officer (24-hour cover by ≥1 medical officer/clinical officer dedicated to the neonatal unit) Registered nurse (1:5/6 patients) (a ratio of one nurse to five newborns was recommended, but in line with recommendations from the LINC example (42), a ratio of 1:6 was deemed acceptable) Nutritionist^1^	Paediatrician (one on call) Medical officer (24-hour cover by ≥1 medical officer dedicated to the neonatal unit) Neonatal nurse Registered nurse (1 nurse of either designation:3 patients) Nutritionist^1^ Counsellor^1^	Neonatologist^1^ Paediatrician (need ≥1 covering the ward 24/7) Medical officer (no consensus on numbers) Neonatal intensive care nurses Neonatal nurses (1 nurse of either designation:1 patient) Nutritionist^1^ Counsellors^1^ Physiotherapist^1^ Occupational therapist^1^ Laboratory technician^1^ Biomedical engineer^1^
Available from the hospital to tend to newborns in the unit as needed (≥1 available to cover neonates)		
	Physiotherapist Occupational therapist Laboratory technician Ophthalmologist	Subspecialists such as Cardiologist Paediatric surgeon Neurosurgeon Plastic surgeon Ophthalmologist Anaesthetist Radiographer/radiologist

LINC, Limpopo Initiative for Newborn Care.

As units would be defined by the services they provide, it was decided that services allocated to each category must represent a realistic minimum. Thus newborn units should meet all service category requirements at lower levels of the hierarchy before adding services linked to higher categories. This approach also recognises that services are co-dependent and if a service is provided, all monitoring and complementary services required for its provision must be available. For example, ophthalmologists should be available to support newborn units aspiring to be in a category where screening for retinopathy of prematurity is a defining feature.

It was also recognised that emergency services should be provided at lower categories of care such that sick newborns requiring referral can be stabilised and transferred. This requires the availability of appropriate minimum diagnostic and clinical services (spanning both skilled personnel and technologies) at facilities with lower categories of care. This also applies to diagnostic and screening procedures, such as thoracic transillumination or screening for retinopathy of prematurity, which would require referral and management at a higher category of care.

## Reflections

Global initiatives such as the Every Newborn Action Plan are prompting countries to focus on newborn care.^52^ Kenya has no specific strategy to guide development of the facility-based neonatal services needed to close access and quality gaps,^14 15 53^ a situation we suspect is common in many LMICs. Developing such strategies and maintaining a pragmatic focus are implicitly also about setting standards and priorities. Group members, and additional experts making a critique of the group’s emerging proposals on behalf of the public sector (see box 1), were fully cognisant of these broader issues.

We based our approach on the SUPPORT framework. However, we deviated from this structure in constraining the options presented for discussion, drawing on prior and detailed knowledge of the context, key findings from the literature and detailed exploration of similar strategies developed in two other LMICs. In this sense, the research group supporting the decision-making process was not an external, objective voice but a partner bringing detailed local knowledge and, arguably, their own biases to the deliberative discussions.

The research team has a long history of constructing panels representative of multiple constituencies to draft paediatric policy recommendations in Kenya.^44^ Engaging contributors who have taken part in earlier evidence-informed decision-making exercises permits familiarity with the group’s work and its understanding of the local context and realities. This may have helped the research team build trust in the process and the synthesised evidence that informed discussions. The engagement of multiple decision-makers over the course of a long-term project may also improve buy-in and the likelihood that recommendations are adopted into policy and practice.^14^

The participants prioritised pragmatic application of their recommendations to the current system over aspirational recommendations for the Nairobi City County context, with a focus on developing recommendations that could be implemented in a 5-year time frame. For example, paediatric radiologists are only found in tertiary centres, and thus it was accepted that a non-specialist radiologist could perform basic gastrointestinal contrast studies. Thus, this service was categorised in the intermediate rather than the intensive category.

Despite this overarching pragmatism, some elements of the guidance tended towards aspirational, such as the recommendation that cranial ultrasound and screening for retinopathy of prematurity should be available at the intermediate category of newborn care. These services are not routinely available in the public sector outside the existing tertiary hospital that provides the only public dedicated neonatal intensive care setting in Nairobi City County. Providing screening and diagnostic procedures at lower categories of care necessitates the concurrent strengthening of the referral system to ensure that the identified patients receive the required management.

Even in the tertiary facility, there is limited availability of additional subspecialists such as paediatric nephrologists and the implicit expectation is that paediatricians with additional neonatal training would often manage peritoneal dialysis. Expanding access to incorporate these more aspirational elements will require specific training, capital investment and ongoing financing. These requirements warrant further discussion in their own right and careful thinking on the tightly related topics of which specialist services may be shared (eg, specialist ophthalmology) and what implications there are for planned referral systems.

Failure to reach consensus on the appropriate categorisation of the Coombs test (standard vs intermediate care) and head cooling (intermediate vs intensive care) also reflected this tension, especially as head cooling is not yet available even in the national referral hospital. These discussions point to a challenge with such decision-making processes in LMICs: the time and expertise available to prepare a comprehensive summary of evidence and for detailed discussions on newer interventions that might benefit from additional expertise is often limited. While the nominal group technique and multiple rounds of engagement ultimately resulted in consensus on the categorisation of the majority of services, efforts to promote consensus-based decision-making did not entirely overcome the potential influence of high-status experts. A further limitation applicable to many LMICs is the absence of information on the potential cost implications of newer interventions.

It was clear to participants that acting on these recommendations will require investment and that ‘business as usual’ will not deliver the required improvements in Nairobi’s neonatal mortality levels. However, the defined categories could help prioritise new investment.

## Next steps

Further discussions are needed to refine, revise and cost recommendations and to build on Ministry guidance for the numbers of staff required at each facility level.^22 53 54^ Developing patient representatives to give feedback in order to take into account the patient and community perspective in these discussions could help to strengthen the recommendations further. The demand for specialised nurses will also need to be addressed as currently there is very limited capacity to provide specialist neonatal nurse training. Infrastructure implications suggest the need for adaptation of existing Ministry recommendations for facility structure^55 56^ providing more granular recommendations applicable to newborn units.

A limitation of this work was that full family-centred care and the particular resources that would be needed to provide it (such as counsellors or accommodation for lodger mothers) was not a focus. Recommendations from WHO, India and South Africa include space allocation for families and family-centred activities.^36 41 57^ India has also developed evidence for empowering families in the care of newborns and alleviating the pressure on healthcare staff.^58^ Further discussions in Kenya would need to incorporate this existing work in order to create a holistic service that responds to the needs of both families and the health system.

For Nairobi, refined recommendations could be mapped onto existing facilities to categorise current system capacity, a step supported by the Investment Framework for Reproductive, Maternal, Newborn, Child and Adolescent Health in Kenya.^59^ With further work, Murphy *et al*.’s estimates of demand^14^ could be stratified by category of newborn care to examine the gap between services required and those available. As much of the private and not-for-profit sectors comprise either small facilities providing poor quality care or larger facilities unaffordable to the majority of the population, focusing on the public sector is a pragmatic approach to achieving equitable, universal effective coverage. Of the four public facilities currently providing 24-hour inpatient neonatal care in Nairobi, three (levels 4 and 5) would correspond at present with the proposed intermediate category and the single tertiary hospital (level 6) would correspond to the intensive care category. However, all would need further upgrading to meet all the requirements of these categories, particularly in the area of staffing.^16^

A key finding from this exercise and earlier work examining existing capacity in Nairobi^15–17^ is that the largest gap in the public sector is provision of the standard category of neonatal care. There are no public facilities offering 24-hour standard category neonatal services that might decongest the four existing, overcrowded public hospitals. This represents a clear disconnect between thinking on EmONC and inpatient neonatal services. There are 217 level 2 and 3 facilities across the public sector in Nairobi, which according to policy should provide 24-hour EmONC services.^22^ However, few are prepared to provide all seven basic signal functions.^21^ Strengthening some of the larger, strategically positioned facilities already providing 24-hour EmONC services to additionally provide 24-hour standard category inpatient neonatal care might allow these units to manage uncomplicated neonatal illness adjacent to maternal services. They could then refer more complex cases and receive convalescing newborns to decongest higher-level units and enable care closer to families’ homes.

A strategy of some decentralisation of standard and intermediate category newborn care linked to regionalisation of intensive category services with concomitant improvements in the referral system could help convert Kenya’s high-level policy goals into tangible changes in the system and address substantial effective coverage gaps in Nairobi City County.^16 17^ This is endorsed by existing Kenyan policies,^7^ which prioritise newborn care with commitment to address key gaps to improve newborn survival.^20^ But with only 7% of the Kenyan health budget allocated to improve quality of care though capital investment^60^ and financial allocations to health forming a barrier to improving staff numbers, improving service delivery to newborns will require effective advocacy and alignment of multiple stakeholders’ efforts. If strategic investments are made, their impact requires local evaluation given the paucity of evidence on organisational change, especially in LMIC contexts.

## Conclusion

There is an ethical imperative to ensure policy is based on the best available evidence,^60^ but changing health policy is a complex process dependent on context.^50^ Limited resources to support evidence-informed decisions in LMICs may mean decision-making processes cannot achieve the increasingly rigorous requirements set in higher-income contexts, limiting their ability to develop recommendations that both meet international standards and fit within contextual constraints.^25^ While the process we describe has limitations, it followed a structured approach, was embedded in the local context and was relatively inclusive and transparent, producing contextually relevant, pragmatic recommendations where none previously existed. This approach could feasibly be replicated in other resource-limited contexts to adapt WHO guidelines for greater specificity and develop locally owned recommendations.

Further improvements in evidence-informed policy-making in such contexts will depend on support for longer-term research and policy-maker partnerships that go beyond individual research grants. Ideally, such efforts should be linked to longer-term evaluation of the implementation of decisions.
