# Supplementary material for: Developing recommendations for neonatal inpatient care service categories: reflections from the research, policy and practice interface in Kenya
Source: BMJ Glob Health. 2019 Mar 7;4(2):e001195. doi: 10.1136/bmjgh-2018-001195 (PMC6441269; doi:10.1136/bmjgh-2018-001195)
Supplement: Supplementary data [file bmjgh-2018-001195supp001.pdf]

SUPPLEMENTARY FILE: APPENDIX

Table A1. Table of documents providing guidance on newborn care in Nairobi County, Kenya

Document	Description	Guidance on Newborn Care
Kenyan Essential Package for Health 2014 (12)	A translation of the right to the highest attainable standard of health (outlined in the Kenyan constitution) into a document designed to give guidance on a basic set of health services provided as close to communities as possible, and aiming for universal access and coverage to comprehensive services. Outlines the hierarchy of levels in the Kenyan Health System – six levels from community to tertiary hospital level.	Guidance on services that should be provided for maternity and child health, including prevention of mother to child transmission of HIV, immunisation, deworming, and management of pneumonia, malaria and diarrhoea.<br>Care for the newborn (services not elaborated) should be provided from level 3 facilities upwards, and inpatient paediatric care from level 4.
Kenya Vision 2030 (62)	National long-term policy aiming to evolve Kenya to a middle-income country that provides a high quality of life to citizens. It is made up of Social, Political and Economic Pillars.	The health sector falls under the social pillar<br>Health indicators for infant and under-5 mortality
Kenya Health Policy 2014-2030 (63)	Provides direction for improvements in health in-line with the constitution, the objectives of devolution and the Vision 2030, outlining the six key objectives of health policy until 2030. It outlines the six levels of the health service delivery system.	Identifies newborns as one of the populations for the objective of provision of essential healthcare
Kenya Health Sector Strategic and Investment Plan 2014-2018 (22)	First strategic plan of the Kenya Health Policy 2014-2030, providing the medium term focus for policy objectives, including an increase in access to the essential package of health by at least 90%	Target: reduce neonatal deaths by half, with a target of 15 deaths per 1000 live births in 2017 (not reached).<br>Service delivery focus is on the prevention of mother to child transmission of HIV, perinatal conditions, congenital anomalies and immunisation.<br>Indicators include the percentage of: deliveries conducted by a skilled attendant, newborns with low birth weight and facility-based under-5 deaths. There are no newborn inpatient-specific indicators.
Kenya Reproductive, Maternal, Newborn, Child and Adolescent Health (RMNCAH) Investment Framework 2016 (20)	Presentation of a set of prioritised interventions for investment and scale up over a five year period to rapidly improve health outcomes.	Neonatal mortality rate is a key indicator.<br>The high impact interventions relevant for newborns proposed by the report are Kangaroo Mother Care, support for feeding preterm babies, management of jaundice, neonatal resuscitation and supportive care for neonatal sepsis.<br>Neonatal-related immediate actions to improve service are bundled with maternal health
Service Availability and Readiness Assessment Mapping 2013 (64)	Health service census mapping the availability of services outlined by the Essential Package for Health, the capacity (human resource, infrastructure and health product availability) and the readiness for the provision of these	The census covered maternal and child health, but focused on prevention of mother to child transmission of HIV, immunisation and services relevant to older children (such as zinc and vitamin A provision).

	services.	The availability of newborn services was measured (30% of primary care facilities and 46% of county hospitals provided the service) but it was not clear what these entailed.
Health Facility Readiness to Provide Emergency Obstetric and Newborn Care in Kenya 2014 (21)	The government of Kenya and USAID partnered to scale up emergency obstetric and newborn care (EmONC) across 15 counties. An assessment was made in 2014 of the availability of EmONC-trained healthcare workers, equipment and medications required to provide the nine EmONC signal functions (seven basic and two comprehensive).	Only one of the nine signal functions is related to neonatal care (ability to perform basic neonatal resuscitation, in the basic EmONC list), assessed as the availability of paediatric bag mask ventilation devices. The other signal functions are concerned with maternal and antenatal care.<br>In Nairobi County, 11% of hospitals and 14% of lower level facilities were assessed as having the full capacity (equipment and commodities) to provide the seven basic EmONC signal functions
Kenya Quality Model for Health Checklist (45)	Conceptual framework to guide facility self-assessment of the organisation of health services, address quality issues and standardise quality improvement initiatives based on evidence and consideration of cultural context. The components are divided into structure, processes and results, with neonatal care forming one of the processes to be assessed.	Checklist covering services that should be provided from level 2: neonatal resuscitation, basic care of the newborn (including prevention of mother to child transmission of HIV, discharge timelines and health education) and the prevention and treatment of sepsis. However, it does not cover other inpatient neonatal services.
Basic Paediatric Protocols 2016 (46)	Clinical management handbook for clinicians and health workers managing newborn and child illness. Targeted at basic hospital care rather than tertiary or university hospitals, but guidance is condition-specific rather than level-specific.	Guidance on the clinical practice of newborn resuscitation, feeding, medication and the management of sepsis, prematurity, low birth weight and jaundice (including exchange transfusion). Provides an indication of the services that should be available at hospitals providing basic inpatient newborn care.
National Guidelines for Quality Obstetrics and Perinatal Care (47)	Reference manual for clinicians to manage conditions associated with maternal and newborn patients with evidence-based interventions.	Covers care of the normal neonate, breastfeeding, resuscitation, birth injuries, management of high risk neonates and management of neonatal emergencies.
Kenya Health Sector Referral Strategy and Implementation Guidelines 2014 (6,7)	Outlines the six levels of the health system and how patients, expertise, specimens and patient information should move between them, as well as the challenges that face the referral system	No newborn specific recommendations
Human Resources for Health Norms and Standards Guidelines for the Health Sector (55)	Guidance on the minimum and appropriate mix of human resources required at each level of the health system	Provides numbers of healthcare staff required by cadre and by facility level for paediatrics, but there is no recommendation for newborn-specific inpatient services.

Table A2. Table showing detailed information on the division of neonatal care in India into three categories of inpatient care and one level of routine care associated with four facility levels

	**Newborn Care Corner (1)**	**Neonatal stabilisation Unit (1)**	**Special Care Newborn Unit (1)**	**Neonatal Intensive Care Unit**
Associated hospital level	Primary Health Centre and hospitals: in the labour room	Level 1 care<br>Community/ Urban Health Centre (36)	Level 2 care<br>District hospital: some in remote districts(36)	Level 3 care<br>Regional perinatal centres and apex institutions
**ADMISSION CRITERIA**				
Age and weight	No admission	Small newborns >1800g<br>>34 weeks gestation (39)	Small newborns 1200-1800g<br>30-34 weeks gestation (39)	Small newborns <1200g<br><30 weeks gestation (39)
Illness	Well newborns (36) – no admission	Sick newborns<br>Referral of sick newborns from Primary Health Centres (36)	Sick newborns (except those requiring mechanical ventilation or surgery (36))<br>• Birth asphyxia (38)<br>• Jaundice (38)<br>• Sepsis (38)<br>• Low birth weight (38)<br>Inborn and out-born babies (38)<br>Down-referred recuperating newborns	Sick newborns requiring mechanical ventilation or surgery (36)
**SERVICES OFFERED**				
Resuscitation	Yes (36)	Yes (36)	Yes (36)	Yes (36)
Routine care at birth	Post-natal care, warmth, weighing, prevention of infection and early breastfeeding initiation (39)		Post-natal care, warmth, weighing, prevention of infection and early initiation of breastfeeding (39)<br>Immunisation (36)	
Medical (1)	Identification and referral of 'at-risk' and sick newborns (3)	Stabilisation of most small and sick newborns<br>1. Jaundice: Phototherapy<br>2. Management of sepsis (37)<br>3. Monitoring of vital signs<br>4. Care of LBW newborns (≥1800g) with no other complications, not requiring intensive care (37)<br>5. Stabilisation and referral of sick newborns or VLBW (37)	Services to manage<br>1. Management of sick newborns ≥1800g<br>2. Birth asphyxia or meconium aspiration: respiratory support (no ventilation) (38)<br>3. Jaundice: phototherapy and exchange transfusion<br>4. Sepsis: intravenous antibiotics (38)<br>5. Low birth weight <1800g<br>6. Gavage feeding<br>Follow up of all babies discharged from the unit	Mechanical ventilation (36)<br>(extent depends on the hospital level (40))<br>Blood bank support (40)<br>Arterial blood gas and electrocardiogram monitoring, with or without invasive monitoring depending on the hospital level (40)
Surgical	Nil	Nil	Nil	Surgical services (36)
Respiratory support	Nil	No information	No mechanical ventilation (36)	Mechanical ventilation (36)
Diagnostic imaging	Nil	No information	No information	Imaging (complexity depends on hospital level, but a minimum includes x-ray and ultrasound) (40)
Pathology	Nil	No information	No information	Laboratory services (complexity depends on hospital level, but a minimum includes basic lab tests such as complete blood count and biochemistry) (40)
**RESPONSIBILITIES**				
Education and training	No information	No information	Training for medical officers and nurses in neonatal care (36)	Training programs in neonatology for medical officers and nurses (36)<br>Research (40)
Community	Community mobilisation, demand generation, support for outreach (36)	Behaviour change, outreach (36)	No information	No information
**INFRASTRUCTURE**				
Space required	1.9-2.8 sq. m in total (36)	3.7-4.6 sq. m /bed<br>18.6 sq. m in total (36)	Minimum of 9.3 sq. m of clear floor space (excluding hand washing stations and columns) per newborn<br>• Baby care area:4.6 sq.m/bed<br>• General support and ancillary areas: 4.6 sq. m /bed	Minimum of 9.3 sq. m /bed (36)

Table A3. Table showing detailed information on the division of newborn care by the LINC project into six categories of care combined into newborn units across the five South African facility levels

	**Routine Care**	**Kangaroo mother care**	**Standard inpatient care**	**High care**	**Intensive care**	**Highly specialised care**
Associated hospital level	All levels of care	Level 1, 2 and 3	Level1,2 and 3	Level 1, 2 and 3	Level 2 and 3	Central hospitals (level 4)
ADMISSION CRITERIA						
Age and weight	Full term, >2000g, (41,42)	Low birth weight <2000g (41,42)	Low birth weight 1500-1999g<br>32-36 weeks gestation<br>>4000g (41,42)	Low birth weight <1500g<br><32 weeks gestation (41,42)	No specific admission criteria by size or age	No specific admission criteria by size or age
Illness	Well newborns (41,42)	Medically stable<br>Down-referral once stabilised in standard, high or intensive care (41,42)	Low Apgars<br>Congenital abnormalities<br>Meconium staining<br>Wasting<br>Possible infection<br>Jaundice<br>(41,42)	Encephalopathy<br>Meconium aspiration<br>Recurrent apnoea<br>Moderate to severe respiratory distress<br>Convulsions<br>Severe infection (septicaemia or meningitis)<br>Severe jaundice<br>Simple surgical conditions (41,42)	Need for assisted ventilation<br>Persistent hypoglycaemia<br>Cardiovascular conditions<br>Multisystem conditions<br>Conditions requiring specialist intervention (41,42)	Need for assisted ventilation<br>Persistent hypoglycaemia<br>Cardiovascular conditions<br>Multisystem conditions<br>Conditions requiring specialist intervention<br>Complex surgical conditions (41,42)
SERVICES OFFERED						
Resuscitation	Basic (41,42)	If required, followed by referral (41,42)	Yes (41,42)	Yes (41,42)	Yes (41,42)	Yes (41,42)
Care	Assessment and measurement<br>Initiation of breastfeeding and support<br>Warmth<br>Vitamin K,<br>Eye care<br>Immunisation (polio and BCG)<br>Cord care<br>PMTCT/ TB/ syphilis prophylaxis<br>Triage to refer LBW, high risk or ill newborns<br>Emergency care and referral<br>Initiating KMC (41,42)	Warmth, stability<br>Nutrition (breastfeeding)<br>Infection prevention (41,42)	Care for the most common illnesses (41,42)<br>Monitoring and correcting glucose<br>Thermal support<br>IV fluid and medication<br>Tube feeding<br>Monitoring bilirubin and phototherapy<br>Drug administration (41,42)	Cardiorespiratory monitoring<br>Blood transfusion<br>Exchange blood transfusion<br>Chest drain<br>(41,42)	Total parenteral nutrition<br>Arterial catheterisation<br>Therapeutic cooling<br>Advanced neurological monitoring<br>Sub-specialist consultation<br>(41,42)	Total parenteral nutrition<br>Arterial catheterisation<br>Therapeutic cooling<br>Advanced neurological monitoring<br>Sub-specialist consultation<br>(41,42)
Respiratory support	Resuscitation then referral (41,42)		Oxygen administration and monitoring<br>(41,42)	Oxygen >40% with a head box<br>Nasal prong CPAP<br>Short term IPPV<br>(41,42)	Intermittent positive-pressure ventilation<br>Advanced respiratory support<br>(41,42)	Intermittent positive-pressure ventilation<br>Advanced respiratory support<br>(41,42)
Surgical	Nil	Nil	Nil	Nil	Neonatal surgical intervention (41,42)	Complex surgical intervention (41,42)
Diagnostics	No information	No information	No information	No information	Ultrasound<br>Echo-cardiography<br>Sophisticated diagnostic investigation (41,42)	Complex investigations (41,42)
INFRASTRUCTURE						
Space required	No information	7.2 sq. m per mother and baby (42)	6 sq. m /bed (42)	7.2-10 sq. m /bed (42)	10-15 sq. m /bed (42)	10-15 sq. m /bed (42)

Table A4. Table of the 38 services for allocation to categories of care, categorised by type of service

**Diagnostics**	**Respiratory support**	**Intravenous access**
Cranial ultrasound	Nasal prong oxygen	Central line
Chest x-ray	Continuous positive airway pressure	Umbilical line
Thoracic transillumination	Invasive ventilation	Arterial line
Upper/lower gastrointestinal barium x-ray	Surfactant	**Laboratory services**
CT/MRI	**Circulatory support**	Full haemogram
**Specific treatment**	Intravenous fluids	Bedside glucose
Intravenous antibiotics	Transfusion of blood (packed red cells)	Lab glucose
Anticonvulsants – intramuscular phenobarbitone	Inotropes	Total bilirubin
Transfusion of blood products (fresh frozen plasma/platelets)	**Feeding support**	Direct bilirubin
Chest drain	Nasogastric tube	Coombs
Peritoneal dialysis	Parenteral nutrition	Urea, Electrolytes, Creatinine
Phototherapy	**Supportive treatment**	**Surgery**
Double phototherapy	Kangaroo Mother Care	Gastroschisis
Exchange transfusion	**Screening**	Imperforate anus
Head cooling	Retinopathy of prematurity screening (for referral to specialist care for management)	Necrotising enterocolitis

Table A5. Table of workshop participants, their background expertise and institutional affiliations.

**Names**	**Background**	**Affiliation**
Participants		
Dorothy Agedo	Nurse educator	Kenyatta National Hospital School of Nursing
Dr Celia Wanda Muturi	Paediatrician	Mama Lucy Hospital
Dr Ester Ogola	Paediatrician	Pumwani Hospital
Francis Muma	Head Quality Assurance Unit	Ministry of Health Department of Standards, Quality Assurance and Regulations
Leah Jepchumba Rutto	County Neonatal Child and Adolescent Health Coordinator	Nairobi County Ministry of Health
Lister Onsongo	Nurse educator	Kenyatta University School of Nursing
Margret Mbaire	Secretary of the Midwives Association of Kenya	Kenya Medical Training College
Mary Kamau	Nurse	Coptic Hospital
Maryline Chebii	Nursing officer	Nursing Council of Kenya
Facilitators		
Jalemba Aluvaala	Paediatrician	KEMRI-Wellcome Trust
Claire Keene	Medical doctor	KEMRI-Wellcome Trust
Observers		
Joyline Jepkosgei	Environmental Health	KEMRI-Wellcome Trust

Table A6. Table describing the modified nominal group process of consensus for allocation of the 38 services to categories of inpatient neonatal care

Round of voting	Number for allocation	Number unanimously allocated	Services allocated
1. Individual allocation by ticking the preferred category in a table of services	38	12	**Standard category**: nasal prong oxygen, phototherapy, Kangaroo Mother Care, nasogastric tube feeding, full haemogram, bedside glucose, total bilirubin, Urea, Electrolytes, Creatinine, intravenous antibiotics, intramuscular phenobarbitone<br>**Intermediate category**: double phototherapy, central line
2. Facilitated discussion of services with one dissenting voter	26	3	**Standard category**: chest x-ray, intravenous fluids<br>**Intermediate category:** umbilical line
3. Individual voting with coloured post-its after discussions and presentation of outputs to the participants and additional expert participants from concurrent workshops	23	17	**Standard category**: direct bilirubin<br>**Intermediate category**: upper/lower gastrointestinal barium, CT/MRI, continuous positive airway pressure, blood transfusion, blood products, chest drain, exchange transfusion, parenteral nutrition and screening for retinopathy of prematurity<br>**Intensive category**: arterial line, invasive ventilation, inotropes, peritoneal dialysis, all surgery (surgery for gastroschisis, imperforate anus and necrotising enterocolitis)
4. Facilitated discussion and voting with a show of hands	6	4	**Standard category**: thoracic transillumination, lab glucose<br>**Intermediate category**: cranial ultrasound, surfactant
Uncategorised because of lack of consensus	2	2	Head cooling and Coombs test

Table A7. Table describing the reasoning for potential allocation to different categories of care for services with a lack of consensus

Service	Arguments for		
	Standard category of care	Intermediate category of care	Intensive category of care
Head cooling	N/A	Despite having potential for severe adverse events, the benefits outweigh the risks for the population of infants it is intended to manage (birth asphyxia), and with the high volume of asphyxiated infants presenting for care in this context, the intensive care category would not be able to meet the need for management of these infants. It was suggested that it would be realistically more beneficial to offer it at an intermediate category and attempt to increase monitoring, than to have asphyxiated infants go without the service.	The serious nature of potential complications means that higher category monitoring and services to manage the complications are required
Coombs test	The supporters of assigning it to standard care argued that it should be a standard for all Rhesus negative mothers, and that it is a useful tool to triage patients and pre-empt danger. It was also argued that if a laboratory can provide haemograms and bilirubin testing, it should have the capacity to provide a Coombs test, and that a holistic package would include all neonatal care-related tests.	Whether the Coombs added value above what is already available was a critical point. The argument for intermediate category allocation was that a Coombs test would not change management, and that there are other tests that had already been allocated to standard care that could be used for management decisions, such as direct bilirubin, rendering the Coombs unnecessary for clinical decision making and more useful for establishing a cause than deciding on treatment. It was further argued that a Coombs would not be performed often enough to warrant providing it at a standard category of care, and that it would be unreasonable to deny a unit the title of a standard category of care because they did not offer a Coombs test	N/A
